# Dietary administration of cumin-derived cuminaldehyde induce neuroprotective and learning and memory enhancement effects to aging mice

**DOI:** 10.18632/aging.202516

**Published:** 2021-01-20

**Authors:** Zineb Omari, Sasaki Kazunori, Mouad Sabti, Meriem Bejaoui, Abdellatif Hafidi, Chemseddoha Gadhi, Hiroko Isoda

**Affiliations:** 1 Alliance for Research on the Mediterranean and North Africa (ARENA), University of Tsukuba, Tsukuba, Ibaraki 305-8572, Japan; 2 Faculty of Sciences Semlalia, Cadi Ayyad University, Marrakesh 40000, Morocco; 3 Open Innovation Laboratory for Food and Medicinal Resource Engineering, National Institute of Advanced Industrial Science and Technology (AIST) and University of Tsukuba, Tsukuba, Ibaraki 305-8572, Japan; 4 Faculty of Pure and Applied Sciences, University of Tsukuba, Tsukuba, Ibaraki 305-8571, Japan; 5 Tsukuba Life Science Innovation Program (T-LSI), University of Tsukuba, Tsukuba, Ibaraki 305-8577, Japan; 6 Faculty of Life and Environmental Sciences, University of Tsukuba, Tsukuba, Ibaraki 305-8587, Japan

**Keywords:** cuminaldehyde, aging, neuroprotection, memory, gene expression

## Abstract

Cuminaldehyde (CA) is one of the major compounds of the essential oil of *Cuminum cyminum*. The aim of this study was to evaluate the effects of CA on aging, specifically on spatial learning and memory. To achieve our objective, an *in vitro* study on SH-SY5Y cells was performed to analyze the neuroprotective effect of CA against dexamethasone using the MTT assay. An *in vivo* study was performed for evaluation of the spatial learning and memory using Morris water maze (MWM). RT-PCR was performed to quantify the expression of specific genes (*Bdnf, Icam* and *ApoE*) in the mice brain. The results obtained showed a neuroprotective effect of CA against dexamethasone-induced neuronal toxicity. The escape latency of CA-treated aged mice was significantly decreased as compared to the water-treated aged mice after 4 days of training in MWM. Moreover, CA treatment up-regulated the gene expression of *Bdnf*, *Icam* and *ApoE*, while it down-regulated the gene expression of *IL-6*. These findings suggest that CA has a neuroprotective effect, as well as a spatial learning and memory enhancement potential through the modulation of genes coding for neurotrophic factors and/or those implicated in the imbalance of neural circuitry and impairment of synaptic plasticity.

Cuminaldehyde (CA) is one of the major compound of the essential oil of *Cuminum cyminum*. The aim of this study was to evaluate the effects of CA on aging, specifically on spatial learning and memory. To achieve our objective, an *in vitro* study on SH-SY5Y cells was performed to analyze the neuroprotective effect of CA against dexamethasone using the MTT assay. An *in vivo* study was performed for evaluation of the spatial learning and memory using Morris water maze (MWM). RT-PCR was performed to quantify the expression of specific genes (*Bdnf, Icam* and *ApoE*) in the mice brain. The results obtained showed a neuroprotective effect of CA against dexamethasone-induced neuronal toxicity. The escape latency of CA-treated aged mice was significantly decreased as compared to the water-treated aged mice after 4 days of training in MWM. Moreover, CA treatment up-regulated the gene expression of *Bdnf*, *Icam* and *ApoE*, while it down-regulated the gene expression of *IL-6*. These findings suggest that CA has a neuroprotective effect, as well as a spatial learning and memory enhancement potential through the modulation of genes coding for neurotrophic factors and/or those implicated in the imbalance of neural circuitry and impairment of synaptic plasticity.

## INTRODUCTION

Every living being undergoes aging. The process of aging affects the brain size, vasculature, cognition [1], and genes, through a constant change in structure from birth throughout the lifetime. In addition, loss of memory also occurs with aging, and brain activation becomes more two aspects for memory tasks [2].

In the present study, we focused on a few genes associated with the loss of memory, brain derived neurotrophic factor (*Bdnf*), interleukin 6 (*IL-6*), apolipoprotein E (*ApoE*), and intercellular adhesion molecule (*Icam*). *ApoE* is an important gene for normal brain lipid homeostasis [3, 4] and is involved in facilitating the availability of cholesterol for development, maintenance and repair of myelin sheaths, nerve membranes, and synaptic connections [5]. *Bdnf* is well known marker for play an important role in long-term potentiation (LTP), a key factor in synaptic plasticity [6, 7]. Cognitive and motivational systems may be affected by neurotoxicity from increased IL-6 expression in the elderly brain [8]. This can also cause Alzheimer’s disease [9]. In addition, increasing age is associated with memory impairment and memory-related dementia; levels of neurotransmitters such as dopamine, noradrenaline, and adrenaline are also altered [2]. For example, dopamine levels have been reported to decrease approximately 10% per decade from early adulthood and have been associated with decreased cognitive function and motor performance [10, 11].

**Figure 1:**
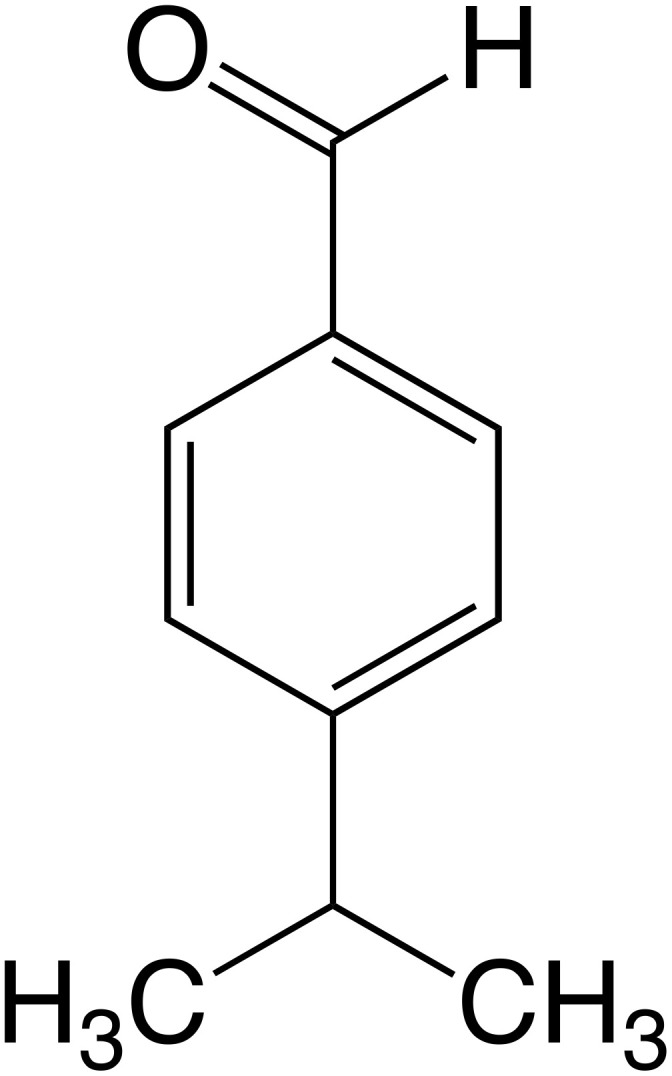
**Structure of cuminaldehyde (CA).**

Cuminaldehyde (CA) (Figure 1) is an aromatic monoterpenoid volatile compound. It is a natural p-isopropylbenzaldehyde, which is one of the activate compound of the essential oil obtained from eucalyptus, myrrh [12], caraway (Carumcarvi) [13, 14], Chinese cinnamon (Cinnamomum cassia), and others [13]. CA also has been reported as the major component of cumin (Cuminum cyminum), a medicinal plant whose seeds are commonly used as a spice in different cuisines. Structurally, CA is a benzaldehyde substituted at the 4^th^ position with an isopropyl group. It is used commercially in perfumes and cosmetics, because it has a pleasant aroma. It has been reported that CA has antidiabetic [15], antitumor [16], anti-inflammatory [17], antimicrobial, and antifungal [18] effects. Recently, studies have shown CA to exert protective effects against neurodegenerative diseases, in particular Parkinson’s disease [19]. However, despite showing great therapeutic potential for the treatment of aging and memory loss in neurodegenerative diseases, no studies have been directed on these effects of CA so far. Thus, the aim of our study is to evaluate the effect of CA on aging, especially on spatial learning and memory using the human-derived neurotrophic SH-SY5Y cells and aged C57BL/6J mice. We also studied the change in expression levels of the associated genes, *Bdnf*, *IL-6*, *ApoE*, and *Icam* in mice brain.

## RESULTS

### Evaluation of the cytotoxicity of cuminaldehyde on human neurotypic SH-SY5Y cells

**Figure 2:**
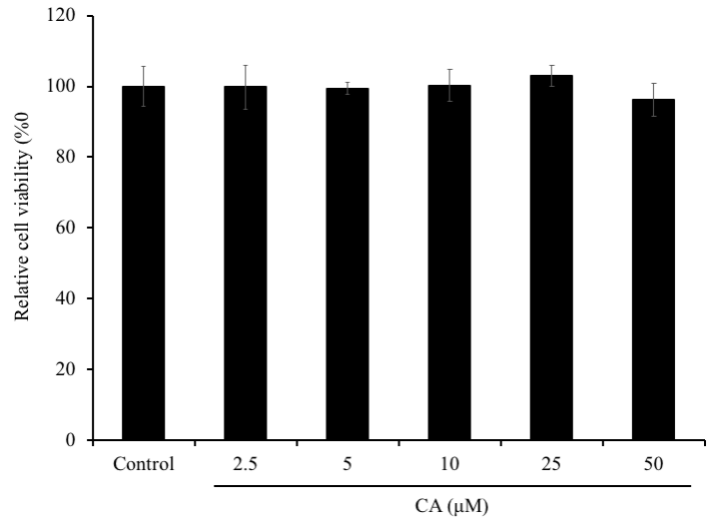
**Effect of CA on SH-SY5Y cells viability.** SH-SY5Y cells were seeded onto 96-well plate at a density of 2×10^5^ cells/well. After overnight incubation, cells were treated with 5, 50 or 100 μM of CA for 72 h and the cell viability was evaluated using MTT assay. The experiment was repeated thrice. The bars signify relative viability as compared to the control. Data is represented as mean ± SD.

First of all, we assessed the effect of CA on SH-SY5Y cells viability using MTT assay. Cells were treated with 2.5, 5, 10, 25, or 50 μM of CA for 72 h (Figure 2). The exposure of the cells to CA for 72 h showed no significant changes in the percentage of the cell viability compared to the non-treated cells (control group) (P > 0.05). Therefore, CA showed no cytotoxicity on SH-SY5Y cells at varying concentrations.

### Evaluation of the neuroprotective effect of cuminaldehyde against dexamethasone-induced neurotoxicity on SH-SY5Y cells

**Figure 3:**
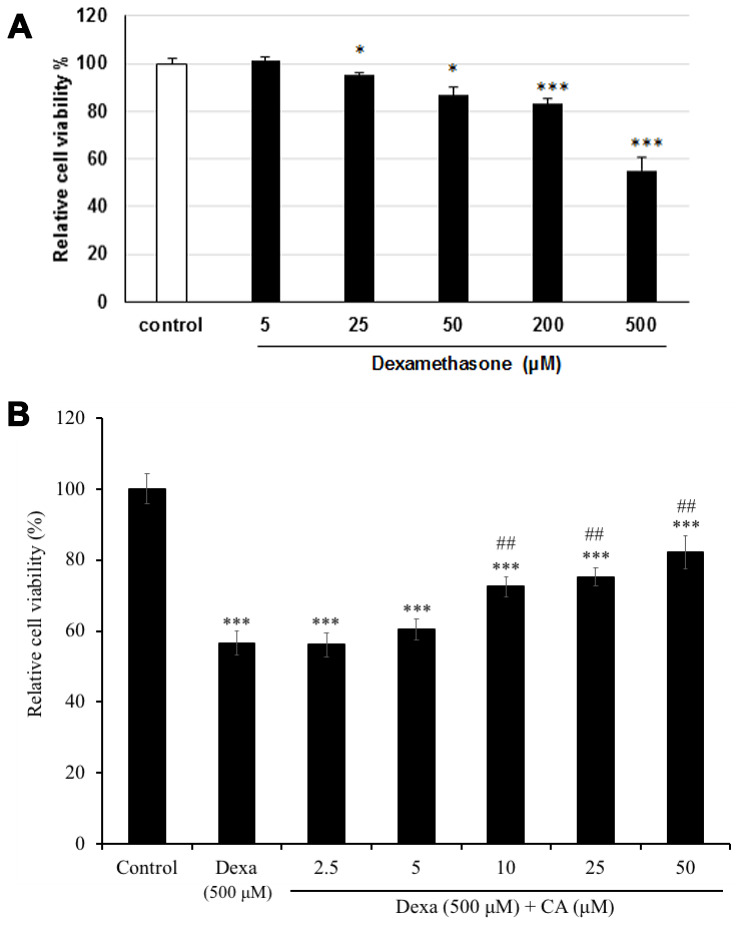
Effect on cell viability on SH-SY5Y cells after treatment with (**A**) dexamethasone (Dexa) and (**B**) Dexa co-treated with CA. SH-SY5Y cells were treated with CA or 500 μM Dexa for 72 h. Each bar represents the mean ± SD. ***: p < 0.001 as compared to the control; ##: p < 0.01; ###: p < 0.001 as compared to Dexa treatment.

SH-SY5Y cells were treated with several concentrations (5, 25, 50, 200, or 500 μM) of dexamethasone (Dexa), to determine the concentration that would be toxic for cells. Dexa-treated cells showed the significantly decrease in the cell viability in a dose-dependent manner, with a decrease of cell viability up to 55% at 500 μM comparing with the non-treated cells (P < 0.001) (Figure 3A). Therefore, the concentration 500 μM of Dexa was used to induce cell cytotoxicity in the further experiments. Then to estimate the effect of CA neurons, SH-SY5Y cells were treated with CA and/or co-treated with Dexa for 72h. The results showed that cell incubated with CA of 10, 25, or 50 μM reversed the Dexa-induced neuronal cell death (Figure 3B), and the cell viability was considerably increased by more than 20% as compared to only Dexa treated cells (P < 0.01).

### Effects of sub-chromic administration of cuminaldehyde on body weight and brain weight of C57BL/6J mice

**Figure 4:**
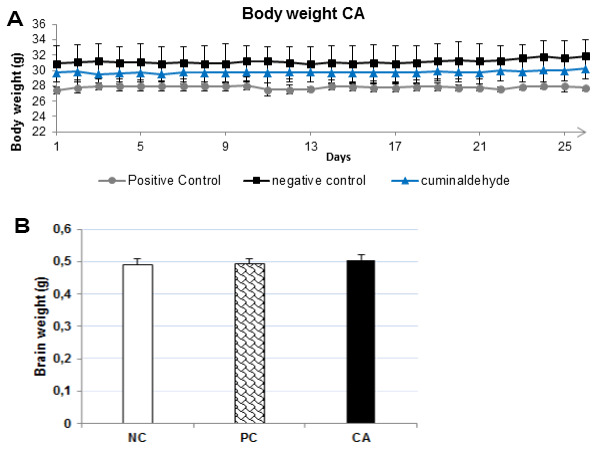
(**A**) Effects of CA (25 mg/kg) on body weight during the 30 days experimental period. All mice were fed a normal diet. The body weight was measured every day. Each point presents the values from that group for that day. The values are represented as mean ± standard deviation. (**B**) Brain weight after 30 days of treatment with CA. The brain was dissected out, washed with ice-cold PBS and weighed. All the values are expressed as mean ± SD. NC: negative control (aged water-treated group); PC: positive control (young water-treated group); CA: Cuminaldehyde treated group.

CA (25 mg/kg) was administered orally daily for 30 days to C57BL/6J mice, and the effect on body weight was studied. The results obtained showed that the body weight of CA treated mice did not differ from the young water-treated mice throughout the experiment (P > 0.05) (Figure 4A), no mortality and no changes in behavioral activity were recorded.

At the end of probe test of MWM, all mice were sacrificed by cervical dislocation and the brain was dissected and weighed. The results showed that CA-treated mice brain weight (Figure 4B) did not differ from those of young-water-treated mice (positive control) or aged-water-treated mice (negative control). CA did not produce any significant effect on the mice body and brain weights after daily administration for 30 days. This suggests that CA may not have any toxic effect in C57BL/6J mice.

### Effect of cuminaldehyde on spatial learning and memory in C57BL/6J mice

**Figure 5:**
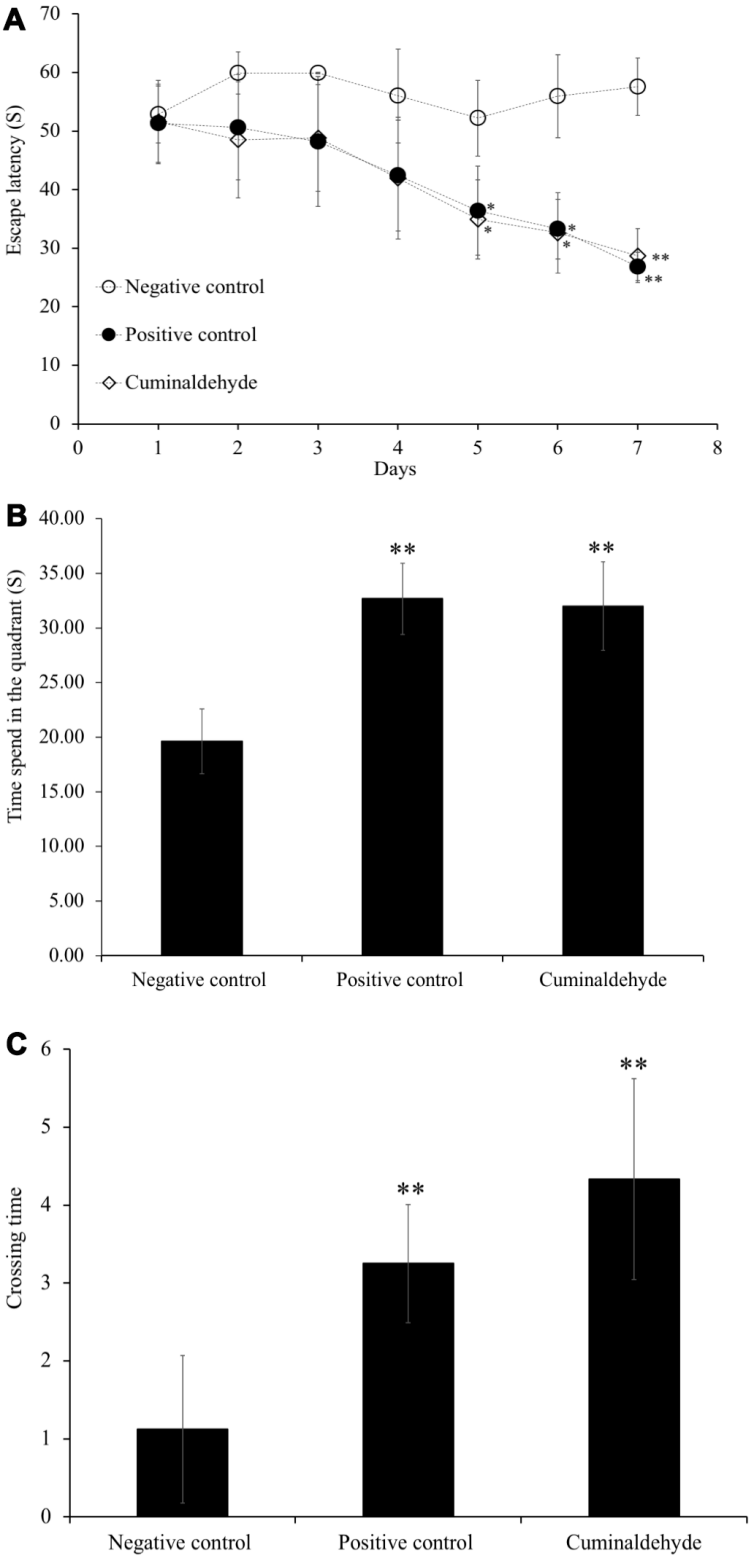
Effects of CA (25 mg/kg) on escape latency time during the morris water maze trial sessions, on (**A**) the time spent in the quadrant and (**B**) crossing time, (**C**) time taken during probe trial sessions on C57BL/6J mice. Mice were administered orally with water or CA (25 mg/kg/day) for 30 days, 60 min prior to trial sessions. The training trial and probe trial sessions were performed as described in the Materials and Methods. All the values are expressed as mean ± SD. NC: negative control (aged water-treated group); PC: positive control (young water-treated group); CA: Cuminaldehyde treated group. * P < 0.05; ** P < 0.01 as compared to the negative control.

To analyze effect CA on the ability of spatial learning and memory in aging C57BL/6J mice, the morris water maze (MWM) test was performed (Figure 5). The time (in seconds) taken by the animal to reach the platform indicates how animal can learn and memorize the platform location. In this experiment, we observed the decrease of escape latency compared to water-treated aging C57BL/6J mice indicating an improvement of spatial learning and memory ability. The escape latency time of both, aged C57BL/6J mice administered with CA (n = 8) and young water-treated C57BL/6J mice (positive control) was significantly decreased by 35% and 31% respectively (P < 0.05) as compared to the water-treated aging C57BL/6J mice (negative control) after 4 days of training session (Figures 4A, 5A). After 7 days of training, the time taken by aged-CA-treated mice (CA group) and young water-treated mice (positive control) significantly decreased by 20% compared to aged water-treated C57BL/6J mice (negative control). On the other hand, there was no significant difference between the escape latency time of aged-CA-treated mice (CA group) and young water-treated mice (positive control).

After 7 days of training, a probe test was performed, in which the platform was removed and the time spent in the quadrant (Figure 5B) and time taken to cross the quadrant (Figure 5C) were evaluated. The results showed that aged-CA-treated mice showed a significant increase by 10% in the time spent in the quadrant (P < 0.01) and the crossing time (P < 0.01) as compared to the aged water-treated mice (negative control).

### Effect of cuminaldehyde on locomotor activity in C57BL/6J mice

**Figure 6:**
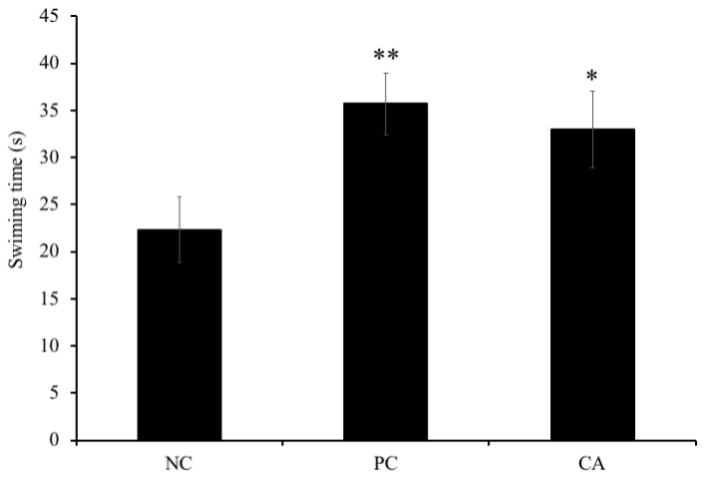
**Effects of CA on swimming time during probe test of morris water maze trial sessions on C57BL/6J mice.** Mice were administered orally with water or CA (25 mg/kg/day) for 30 days 60 min prior to trial sessions. Data is represented as means ± SEM (* P < 0.05 as compared to negative control).

In the probe trial (day 8), we also measured immobility time of mice for evaluation of their motivation. Our results showed that there was a significant increase by 16% (P < 0.05) in the time spent swimming by aged-CA-treated group compared to the aged-water-treated group (negative control), however; no significance was observed between the aged-water-treated mice (negative control) and the young water-treated group (positive control) (P > 0.05) (Figure 6).

### Effect of cuminaldehyde on gene expression in C57BL/6J mice

**Figure 7:**
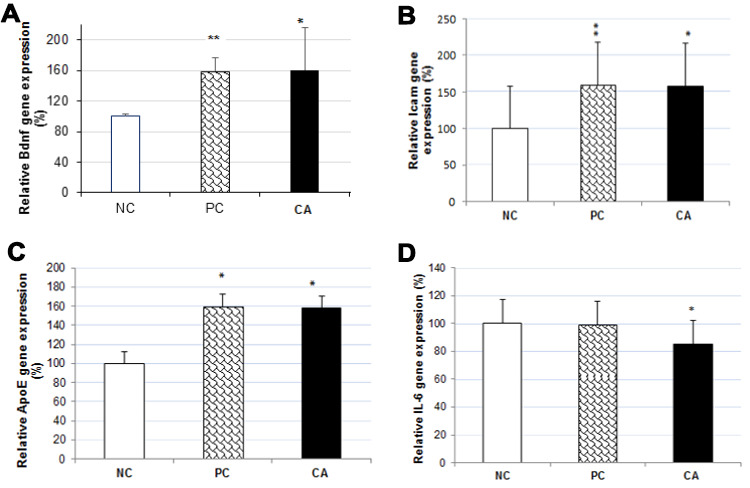
Effect of CA on mRNA expressions of Bdnf (**A**), Icam (**B**), ApoE (**C**) and IL-6 (**D**) in C57BL/6J mice brain. The mRNA expression of all genes were normalized to GAPDH mRNA expression and expressed with respect to the negative control (aged water-treated group). C57BL/6J mice were administrated with CA for 30 days. The control group was administered with distilled water. Each bar represents the mean ± SD. * P < 0.05, ** P < 0.01 as compared to negative control group. NC: negative control (aged water-treated group), PC: positive control (young water-treated group), CA: Cuminaldehyde treated group.

To determine the molecular mechanism underlying the enhancement of spatial learning and memory of CA, we analyzed changes in gene expression of four genes (*Bdnf*, *IL-6*, *Icam*, and *ApoE*) in the mice brain using RT-PCR (Figure 6). Compared to the aged water-treated mice (negative control), a dose of 25 mg/kg/day of CA treatment in C57BL/6J mice, for 30 days, up-regulated the expression of *Bdnf* by 58% (Figure 7A), *Icam* by 34% (Figure 7B), and *ApoE* by 58% (Figure 7C), while it down-regulated the expression of *IL-6* by 15% (P < 0.05) (Figure 7D).

### Effect of cuminaldehyde on neurotransmitters concentration in C57BL/6J mice brain

**Figure 8:**
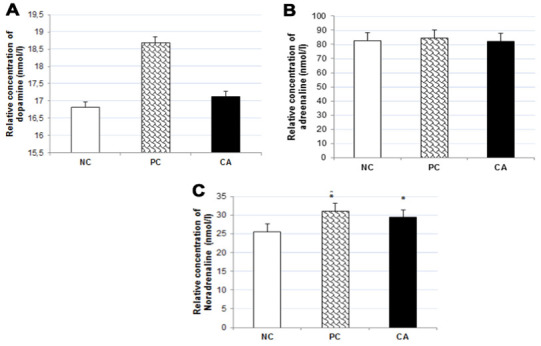
Effect of CA on catecholamine concentrations: Dopamine (**A**), Adrenaline (**B**), and Noradrenalin (**C**) in C57BL/6J mice brain. C57BL/6J mice were administrated with CA for 30 days. The control group was administered with distilled water. Each bar represents the mean ± SD. * P < 0.05, ** P < 0.01, *** P < 0.001 as compared to negative control group. NC: negative control (aged water-treated group), PC: positive control (young water-treated group), CA: Cuminaldehyde treated group.

To evaluate the effect of CA on the levels of dopamine, adrenaline, and noradrenaline in the brain of C57BL/6J mice, their concentrations in the brain were measured using ELISA technique. The results showed no significant changes in the concentrations of dopamine (Figure 8A) and adrenaline (Figure 8B) in CA-treated mice brain compared to aged water-treated mice (negative control); whereas there was a significant increase (13%, P < 0.05,) in the concentration of noradrenaline (Figure 8C).

### Effect of cuminaldehyde on the levels of TNF-α and IL-6 in C57BL/6J mice brain

**Figure 9:**
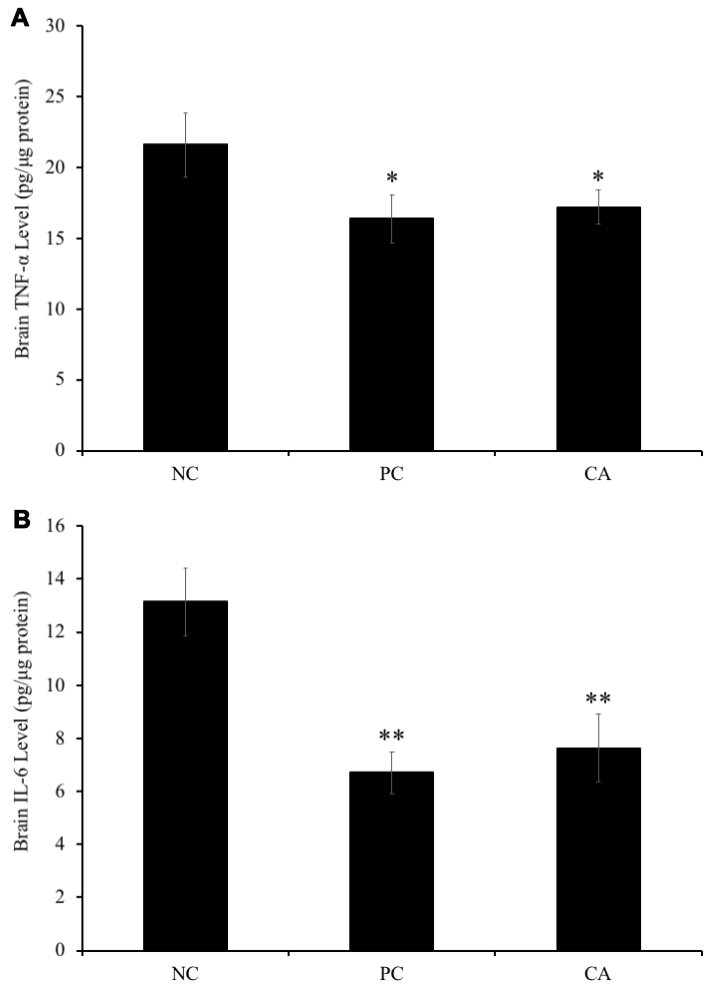
Effect of CA on levels of (**A**) tumor necrosis factor-α (TNF-α) and (**B**) interleukin 6 (IL-6) in C57BL/6J mice brain. C57BL/6J mice were administrated with CA for 30 days. The control group was administered with distilled water. Each bar represents the mean ± SD. * P < 0.05, ** P < 0.01 as compared to negative control group. NC: negative control (aged water-treated group), PC: positive control (young water-treated group), CA: Cuminaldehyde treated group.

To evaluate the effect of CA on the levels of TNF-α and IL-6 in the brain of C57BL/6J mice, their concentrations were measured in the brain using ELISA technique. Our result showed that brain TNF-α was significantly (P < 0.05) increased in aged water-treated mice (negative control) (21.6 ± 2.3 pg/μg protein) compared with young water-treated group (positive control) (16.4 ± 1.7 pg/μg protein) (Figure 9A). However, CA-treated group showed a significant decrease (17.2 ± 1.2 pg/μg protein) of TNF-α levels in brain compared with aged water-treated group (negative control) (P < 0.05, Figure 9A).

Moreover, brain IL-6 level in aged water-treated group (negative control) was significantly (P < 0.01) increased compared with young water-treated group (positive control) (6.7 ± 0.8 pg/μg protein) (Figure 9B). Similarly, IL-6 level in the CA-administered group (7.6 ± 1.3 pg/μg protein) was significantly increased (P < 0.01) as compared to that of aged water-treated group (negative control) (13.1 ± 1.3 pg/μg protein).

## DISCUSSION

The activities of daily life of the majority of elderly people are affected by a form of memory loss. The incidence of dementia and memory impairment rise with the age. In addition, there are changes in the levels of neurotransmitters [2], for example, dopamine level is reported to decline with age and has been associated with decline in cognitive and motor performance [10, 11]. Therefore, any factor that may enhance the memory of elderly people will be of a great importance. CA is one of the major components of cumin, the second most popular spice in the world after black pepper. In the present study, CA was investigated for its neuroprotective effect and its potential in memory enhancement was evaluated.

To evaluate the neurotoxicity and the neuroprotective effect of CA, SH-SY5Y cells were treated with Dexa and/or CA. MTT results showed that the treatment of SH-SY5Y cells to CA for 72 h had no significant effect on the cell viability compared to the non-treated cells (control). All the concentrations of CA showed no cytotoxicity on SH-SY5Y cells; and the highest death rate occurred when Dexa was used alone at a concentration of 500 μM (Figure 3). However, when CA was co-incubated with Dexa, there was a significant decrease in cell death. Our results show that CA has a neuroprotective effect against Dexa-induced neurotoxicity. It has been shown that CA possesses antioxidant activity [20]; therefore, it can be speculated that the increased cell viability could be due to the antioxidant properties of CA.

In the present study, we also assessed the effect of CA on mice at the molecular level by real time PCR for analyzing the alterations in expression of some genes. We used the MWM to evaluate learning memory and locomotor activity. CA was administrated orally every day for 30 days at the concentration of 25 mg/kg, and the body weight was measured every day before oral administration. No mortality was observed and our results show that the body weight did not change throughout the experiment. This suggests that CA is not toxic at the tested concentration. However, unfortunately, there are currently no reports on the pharmacokinetics of CA. Therefore, in the future it is necessary to investigate the pharmacokinetics of CA, especially whether it can cross the blood-brain barrier.

The MWM test, tool for evaluation of spatial learning and memory tasks in rodents, was used in this experiment. Our results show that the escape latency time of aged C57BL/6J mice group administered with CA and young water-treated C57BL/6J mice group was significantly decreased as compared to the aged water-treated C57BL/6J mice from the 4th day of training. After 7 days of training, the escape latency time in aged CA-treated mice (CA group) and young water-treated mice significantly decreased with 20% compared to aged water-treated C57BL/6J mice. On the other hand, C57BL/6J mice administered with CA group show a decrease in the time spent in the quadrant and the crossing time compared to the aged water-treated C57BL/6J mice. Together, all the results obtained show that CA increases learning and memory. In the probe trial, time spent in the quadrant by mice swimming was recorded, and there was a significant increase in time spent swimming between aged CA-treated group and the aged water-treated group. Our findings suggest that CA also increases the locomotor activity in C57BL/6J mice.

Moreover, to understand the mechanism of action of CA and how it affects learning, memory, and locomotor activity, the expression of several genes involved in nerve cells growth and differentiation, neural circuitry, and synaptic plasticity was analyzed. Using RT-PCR, we analyzed the expression of *Bdnf*, *IL-6*, *Icam*, and *ApoE* genes. Our results showed that brain of 30 days CA treatment in C57BL/6J mice, up-regulated the gene expression of *ApoE* by 58% (P < 0.05) as compared to aged water-treated mice group. ApoE is a polymorphic protein synthesized by astrocytes and microglia but it can also be produced by neurons following injury [5]. ApoE is also strongly expressed in brain and liver [3, 4].

Various studies on learning and memory using animal models have identified many genes associated with these processes. Among them is the brain-derived neurotrophic factor (*Bdnf*), which is also a widely studied gene in brain research. Our results showed that brain of 30 days CA treatment in C57BL/6J mice up-regulated the gene expression of *Bdnf* by 58% as compared to aged water-treated group. The role of Bdnf in learning and memory has already been established by studies carried out in *in vivo* rodent models and *in vitro* cell models. Bdnf mRNA expression was reported to be increased in the hippocampus of rats following training with MWM [21], radial maze [22], passive avoidance [23], and contextual fear conditioning [24]. Increased Bdnf expression has been shown to play an important role in LTP, which contributes to synaptic plasticity, a form well-established as a cellular model for long-term memory (LTM) formation [6, 7]. In studies related to synaptic plasticity, LTP is the most studied form and is considered as a cellular correlate of learning and memory. The induction of LTP is associated with activation of multiple signaling cascades and is also activated by Bdnf [25]. In some *in vitro* studies, exogenous Bdnf induced the promotion of the LTP in young hippocampal slices [26], and rapidly increased the frequency of micro-excitatory postsynaptic currents in solitary neurons [27]. LTP and synaptic plasticity are known to be associated with ultrastructural changes in the dendritic spines at excitatory glutamatergic synapses [28–31]. LTP and synaptic plasticity are responsible for enormous ultrastructural changes [26–29] such as the formation of new spines [30–32] and the increase in the number of glutamatergic AMPA receptors at the level of dendritic spines [33–35]. These results suggest Bdnf plays a crucial role in learning and memory processes.

In our study, CA treatment down-regulated both, the gene and protein expression of IL-6 compared to the aged water-treated mice. The expression of IL-6 is under tight regulation in the leukocytes; however, with an increasing age this regulation is relaxed, so the circulating levels of IL-6 are reported to increase with an increase in the age [36, 37]. A recent study in old healthy people by Weaver (2002) reported a modest decline in cognitive ability in an age-dependent manner with increase in plasma IL-6. In addition, the results of gene expression levels showed an increase in *IL-6* mRNA levels in the whole brain of aged mice compared to young adult mice [38]. Levels of *IL-6* in the several brain parts such as hippocampus, cerebral cortex, cerebellum were increased in aged mice compared to young adult mice [39]. Therefore, the increase of gene expression levels of *IL-6* in the aged mice brain is considered potentially relevant, as IL-6 may be neurotoxic and may affect cognitive and motivational systems [8]. This can also cause Alzheimer’s disease [9], in which senile plaques are formed around microglia and astrocytes, considering that the progression of the disease is partially caused by several cytokines and neurotoxic factors [40]. The expression of *IL-6* in astrocytes, microglia, and neurons, which has been suggested to decrease the food intake, regulate the ability of memory and learning, cause neurodegeneration, and exacerbate illness induced by other cytokines in the brain [41]. Our results suggest that CA decreases the level of *IL-6* and thereby, has a protective effect against IL-6 induced memory loss. Our results also showed an increase in the concentration of noradrenaline by 13% in CA-treated mice compared to the aged water-treated mice. During normal aging, noradrenaline level in old animals is significantly lower in the hypothalamus, as compared to the young rats [42]. Stimulation of noradrenergic receptors in the hippocampus results in changes in neuronal excitability and synaptic plasticity, considering an important role for noradrenaline in learning and memory. Consistent with this notion, noradrenaline enhances memory for various hippocampal-dependent tasks. For example, the effect of noradrenaline receptor activation on cellular plasticity may cause noradrenaline-dependent regulation of memory in the hippocampus. In addition, dysfunction of the noradrenergic neuromodulatory system causes many cognitive and mental disorders [43].

On the other hand, our results show no significant changes in the concentration of dopamine and adrenaline compared to aged water-treated mice, which contradicts the literature that reports that the levels of dopamine [44–46] and adrenaline in the rat and mice brain decrease with aging [47, 48]. Dopamine plays an essential role in the control of body movements. Age-related reductions in dopamine levels are considered to be responsible for many age-related neurological symptoms, such as decreased arm swing and increased rigidity [49]. In addition, fluctuations in dopamine levels can cause age-related changes in cognitive flexibility. In our study, we observed an increase in the swimming time in CA-treated mice, which corroborates with the finding that an increase in neurotransmitter level (dopamine) increases the locomotor activity in CA-treated group.

## CONCLUSIONS

In conclusion, the treatment of SH-SY5Y cells with CA shows a neuroprotective effect against Dexa-induced cells toxicity. Moreover, oral administration of CA demonstrated learning and memory enhancement in the MWM test. This effect is co-related to a significant increase in the concentration of noradrenaline; and is also related to the up-regulation of *Bdnf*, *Icam*, and *ApoE* expression and down-regulation of *IL-6* expression that is increased with aging. Our findings provide the first evidence that CA has a neuroprotective effect, as well as a spatial learning and memory enhancement potential through the modulation of genes coding for neurotrophic factors and/or those implicated in the imbalance of neural circuitry and impairments of synaptic plasticity. In the previous study, it should be pointed out that CA accounted for 48.77 % of the whole content of cumin essential oil [36]. Therefore, it may be considered that daily intake of cumin essential oil can contribute to reasonable improvement of cognitive function. Altogether, our results suggest that CA could be a promising drug for elderly against amnesia and memory deficits.

## MATERIALS AND METHODS

### Chemicals and reagents

CA (Figure 1), Dulbecco’s Modified Eagle Medium (DMEM), Ham and Fetal bovine serum (FBS) were purchased from Sigma Aldrich Co., Ltd. (Irvine, United Kingdom). Penicillin and Streptomycin were purchased from Lonza Inc. (Walkersville, MD), while Eagle’s minimum essential medium (OPTI-MEM) was purchased from Gibco (Japan), and 3-(4,5-dimethyl-2-thiazolyl)-2,5-diphenyl-2H-tetrazolium bromide (MTT) from DOJINDO, (Japan). SDS was purchased from GE Healthcare (Sweden).

### Cell culture

The human neuroblastoma SH-SY5Y cell line was obtained from American Type Culture Collection (ATCC). This cell line is widely used in the field of neuroscience for the research on Alzheimer's disease, neurotoxicity, ischemia, and amyotrophic lateral sclerosis [50]. According to our previous study [51], SH-SY5Y neuroblastoma cells were cultured in 100 mm petri dish or in 96-well plates with a 1:1 (v/v) mixture of Dulbecco’s Modified Eagle Medium (DMEM) and Ham’s F-12 nutrient mixture supplemented with 15% fetal bovine serum (FBS), and 1% penicillin (5000 μg/mL) streptomycin (5000 IU/mL) solution at 37° C in a 95% humidified air of 5% CO2 incubator. A serum-free Eagle’s minimum essential medium (Opti-MEM) was used to culture the cells for cell viability assay.

### MTT assay

To assess the cell viability of SH-SY5Y cells, MTT assay was performed as previous our study [51]. SH-SY5Y cells were cultured at a density of 2 x 10^5^ cells/well in a fibronectin coated 96-well micro-plate (BD, BioCoat). After a period of 24 h incubation at 37° C (5% CO), cells were treated with CA, at concentration of 5, 50 or 100 μM, dissolved in Opti-MEM. After 72 h of incubation, 10 μL of MTT (5 mg/mL) dissolved in 100 μL of Opti-MEM was added to each well and the plate was incubated for further 6 h. The colored formazan was dissolved in 100 μL of 10% SDS. The cell viability was determined using a multi-detection micro plate reader (Powerscan_1-42, HT, Dainippon Pharmaceutical, Japan) using absorbance at 570 nm, and the results were represented as percentage compared to control.

### Neuroprotective assay

To investigate the neuroprotective effect of CA, Dexa, a synthetic glucocorticoid used as an inducer of apoptosis, was used according to our previous study [51]. SH-SY5Y cells were cultured in 96-well plate (fibronectin-coated plate) and treated with CA and co-treated with Dexa (500 μM) for 24 h and 72 h. After the treatment, 100 μL of Opti-MEM and 10 μL of MTT (5 mg/mL) were added, and cell viability assay was performed as described above.

### Animals

C57BL/6J male mice aged 10 weeks and 9 months, weighting between 25 g and 35 g were used in the experiment. The animals were obtained from Japan (Japan SLC Company, Japan). Mice were housed individually, one mouse per cage and had free access to food and water, under a 12 h light/12 h dark cycle in a controlled environment of 56% humidity and 23° C temperature.

After 7 days of acclimatization to the laboratory conditions, mice were divided into 3 groups: a positive control group composed of 10 weeks-old mice (young water-treated group) (n = 7), a negative control group composed of 9 months-old mice (aged water-treated group) (n = 5), and CA-treated group composed of 9 months-old mice (n = 8) which were treated by oral administration of CA (25 mg/kg) for 30 days. The concentration dose (25 mg/kg) of CA for *in vivo* study was determined based on the previous study using cuminum cyminum extract [52]. The respective control groups (positive and negative groups) were administered an equivalent volume of distilled water. This animal experiment was approved by the Animal Care and Use Committee of the University of Tsukuba.

### Morris water maze (MWM) test

The MWM test was carried out using a circular pool of 120 cm in diameter and 45 cm in height as reported previously [53]. On the inner surface of the circular pool four symbols (a square, a circle, a cross mark, and a triangle) were attached. The pool was divided into four quadrants (north, east, west, and south) and filled with water (23 ± 2° C). A Plexiglas platform (10 cm of diameter) was placed exactly in the northeast quadrant, submerged 1 cm below the water surface. The platform was fixed in the same place in the pool throughout the experiment. The mice were given a trial session (60 s) 4 times each day for 7 days, then each mice were placed on the platform for 30 s. CA was administered to the treatment group 30 min before the experiment.

After 7 days, each mouse performed one probe trial, in which the platform is removed from MWM pool. Probe trials are performed to validate the strategy followed by the animals when they notice an absence of the platform. The animals were released into the pool from a single point and the number of times the animal crosses the quadrant of the platform in 30 s was recorded.

### Tissue preparation

Mice were sacrificed by cervical dislocation 24 h after the last trial of the MWM test and 30 min after the last oral administration of the pure compound. The brain samples were dissected and fixed in liquid nitrogen, and stored in -80° C until use.

### RNA extraction from mouse brain

A total of 100 mg of the cerebral tissue was removed and washed with ice-cold phosphate-buffered solution (PBS). The total RNA was extracted from it using the ISOGEN kit (Nippon Gene Co. Ltd., Toyama, Japan), following the manufacturer’s instruction. Total RNA was quantified and assessed for its purity with the NanoDrop 2000 spectrophotometer (Thermo Scientific, Wilmington, DE, USA).

### RT-PCR

The Superscript III reverse transcriptase kit (Invitrogen, Carlsbad, CA, USA) allowed us to carry out reverse transcription reactions [54]. Briefly, the RNA was denatured at 65° C for 5 min, incubated with 1 ml of oligo primer (dT) and cooled to 4° C. After adding the Superscript III reverse transcriptase (200 units), the reaction mixture was incubated at 42° C for 60 minutes, then for 10 minutes at 70° C. The cDNA was used to evaluate the gene expression of *Bdnf*, *ApoE*, *Icam*, and *IL-6*. This experiment was conducted using TaqMan Universal PCR mix and TaqMan Probes, and the amplification reaction was performed in a 7500 Fast Real-time PCR (Applied Biosystems, USA) with the following conditions: 50° C for 2 min, followed by 95° C for 10 min, and 40 cycles of 95° C for 15 s followed by 60° C for 1 min.

### Dopamine, noradrenaline and adrenaline levels in mice brain

Dopamine, noradrenaline, and adrenaline levels in the mice brains were measured by Dopamine, Adrenaline, and Noradrenaline Research ELISA kits (ImmuSmol, Pessac, France) as reported in the previous study [54]. Briefly, the cerebral cortex (100 mg) was isolated from brain tissue of mice and homogenized in radioimmunoprecipitation assay (RIPA) buffer with protease inhibitor (Santa Cruz Biotechnology, Inc., Tokyo, Japan). Further, 50 μL of protein samples or 10 μL standards were used for catecholamine extraction, acylation, and determination according to manufacturer’s instructions. After optimal color development, the reaction was stopped and the absorbance was measured at 450 nm. Protein concentration was determined using the 2-D Quant kit (GE Healthcare Inc., Tokyo, Japan) and the data were expressed as ng/μg protein.

### Brain tumor necrosis factor-α (TNF-α) and interleukin 6 (IL-6) quantification

Brain tumor necrosis factor-α (TNF-α) and interleukin 6 (IL-6) levels in the brain were measured using ELISA kit (TNF-α: Proteintech, Japan; IL-6: Invitrogen, Japan) in accordance with the manufacturer’s instructions. Briefly, 100 mg of the limbic area was isolated from the brain tissue and homogenized in RIPA buffer with protease inhibitor (Santa Cruz Biotechnology, inc., Tokyo, Japan). Following centrifugation (1000 × g, 20 min), the supernatants or standards were used to determine the TNF-α and IL-6 levels in brain. After the addition of TNF-α or IL-6 antibody, a second incubation was performed with streptavidin-horseradish peroxidase conjugate solution. After addition of substrate and stop solution, TNF-α and IL-6 levels were determined by measuring absorbance at 450 nm.

### Statistical analysis

All results are expressed as mean ± standard deviation (SD), and statistical analyses were performed using a Student’s t-test using SigmaPlot 12.0 software. Results were considered statistically significant when P-value was less than 0.05.
